# Mitochondria-Targeting Immunogenic Cell Death Inducer Improves the Adoptive T-Cell Therapy Against Solid Tumor

**DOI:** 10.3389/fonc.2019.01196

**Published:** 2019-11-12

**Authors:** Qingzhi Jiang, Chi Zhang, Huilan Wang, Tao Peng, Li Zhang, Yang Wang, Weidong Han, Chunmeng Shi

**Affiliations:** ^1^Department of Oncology, The Affiliated Hospital of Southwest Medical University, Luzhou, China; ^2^State Key Laboratory of Trauma, Burns and Combined Injury, Institute of Rocket Force Medicine, Third Military Medical University, Chongqing, China; ^3^Department of Blood Transfusion, The General Hospital of Western Theater Command, Chengdu, China; ^4^Department of Molecular Biology, School of Life Sciences, Institute of Basic Medicine, Chinese PLA General Hospital, Beijing, China

**Keywords:** tumor targeting, mitochondria, immunogenic cell death, adoptive T-cell therapy, immunotherapy

## Abstract

Cancer immunotherapy including adoptive T cell therapy (ACT) is widely used in the clinic and is highly beneficial for patients with hematological malignancies; however, it remains a challenge to develop effective immunotherapy strategies for the treatment of solid cancers, due to the inefficiency of the immune response and the immunosuppressive tumor microenvironment (TME). Immunogenic cell death (ICD) converts dying cancer cells into a therapeutic vaccine and stimulate a systemic antigen-specific antitumor immune response, which can effectively subvert the immunosuppressive TME and enhance the efficiency of immune responses, relative to conventional immunotherapeutic regimens. However, the application of traditional inducers of ICD in anti-cancer immunotherapy has been limited because of low levels of ICD induction and a lack of tumor-targeting accumulation. Mitochondria are important for tumor-targeting strategies and have emerged as organelles with key roles in the immune system. We hypothesized that the alteration of mitochondria in cancer cells could be an important target for the development of an efficient ICD inducer for use in cancer immunotherapy. Here, we report the evaluation of a mitochondria-targeted small molecule, IR-780, that acts as an ICD inducer and exhibits exceptional antineoplastic activity. IR-780 specifically accumulated in tumor cells to elicit ICD *in vitro* and *in vivo*, effectively suppressed tumor growth and lung metastasis, and enhanced adoptive T-cell therapy effects against solid tumors in mouse models. These anticancer effects were linked to dendritic cell maturation and synergistic effector T cell priming and infiltration into tumors. The underlying mechanism involves the direct targeting of the mitochondria by IR-780, to destroy cancer cells, including drug-resistant cancer cells, leading to the full exposure of tumor-associated antigens (TAAs), thereby enhancing antigen-specific antitumor immune responses. These features of IR-780 suggest that it has the advantage of leading to complete TAA exposure and the stimulation of efficient antitumor immune responses in the TME. IR-780 has potential for use as a preparative ICD inducer, in combination with conventional immunostimulatory regimens for cancer immunotherapy, particularly in the context of solid tumor treatment.

## Introduction

In the last decade, immunotherapy has emerged as the most promising cancer treatment strategy, alongside chemotherapy, radiation therapy, and surgery (1). Immunotherapy enhances the specificity and power of the immune response, to eradicate cancer cells in a variety of human malignancies, resulting in durable antitumor responses, which reduce tumor metastasis and recurrence more effectively than traditional treatments (2, 3). One cancer immunotherapy strategy, using adoptive T cell therapy (ACT), with either allogeneic or autologous immune cells, has shown unequivocal therapeutic benefits in a substantial fraction of cancer patients (4, 5). More recently, ACT has been developed to use genetically engineered T cells, with the introduction of an additional chimeric antigen receptor (CAR) or T cell receptor (TCR) to crosslink target and effector cells. CAR T therapy in particular has been widely used in the clinic and is highly beneficial for patients with B cell malignancies (6); however, it remains a challenge to develop effective ACT strategies for the treatment of solid cancers, due to the inefficiency of effector T cell activation and the immunosuppressive tumor microenvironment (TME), resulting from chronic, but suboptimal exposure to tumor-associated antigens (TAAs) (7). Hence, there has been considerable interest in the development of complementary approaches, including therapeutic chemotherapy, which can fully expose TAAs and stimulate an antitumor microenvironment (8), to induce increased and prolonged adoptive T cell responses at tumor sites, exerting strong antitumor immunity against solidtumors (9).

Notably, recent studies have shown that a special form of cancer cell death, known as immunogenic cell death (ICD) (10), caused by specific types of chemotherapeutic drugs, radiotherapy, or photodynamic therapy, may contribute to antitumor T cell responses. Tumor cells undergoing ICD up-regulate the expression of calreticulin (CRT) on their surfaces, where CRT provides “eat me” and “danger” signals, which induce dendritic cells (DCs) to devour tumor cells and present TAAs. The subsequent release of ATP and high-mobility group box 1 (HMGB1) by tumor cells undergoing ICD promotes DC activation and triggers antigen-specific T cell responses (11, 12). Thus, the use of ICD-inducing agents may offer a convenient strategy for killing cancer cells, while simultaneously eliciting broad antitumor T cell responses (13); however, the immunogenic consequences largely depend on cancer cell targeting therapeutic effects, exposure of TAAs, and the level of the antitumor immune response. The application of traditional ICD inducers in anti-cancer immunotherapy is limited because of their lack of tumor-targeting and accumulation, as well as their weak induction of ICD (14).

Recent reports have emphasized the emergence of mitochondrial alterations as a hallmark of cancer cell biology (15, 16). In addition to their involvement in bioenergetic and biosynthetic processes, mitochondria are integral to stress sensing, which allows cellular adaptation to the microenvironment, and confer vital roles in the regulation of tumorigenesis and tumor progression (17). Many studies have indicated that some types of tumor, or cancer cell subpopulations, rely more heavily on mitochondrial functions. Thus, targeting mitochondria in cancer cells provides an opportunity for development of tumor-targeting treatment strategies, particularly for cancer stem cells or drug-resistant cancer cell subpopulations, which may be more dependent on mitochondria (18, 19). Mitochondria are the only organelles that retain a small DNA genome, which encodes proteins essential for respiration. Cancer cells harbor alterations in mitochondria, including mutations of mtDNA and proteins, which contribute to the cellular adaptation of the complex TME (20). Important roles for mitochondria in regulation of the human immune system have also emerged, including the control of signaling pathways involved in the stimulation of innate and adaptive immunity, as well as the metabolic regulation of immune function and the establishment of immune cell phenotypes (21). These characteristics provide a rationale for targeting aberrant mitochondria and tumor-associated mitochondrial antigens, which meet all the criteria of ideal targets for the effective induction of ICD (22). Therefore, we hypothesize that agents targeting mitochondria could be important candidates for development as efficient tumor-targeted ICD inducers for application in solid tumor immunotherapy. In previous work, we have identified a class of heptamethine cyanine dyes characterized with a tumor-targeted ability and which prefer to accumulate at the mitochondria in a manner that is dependent on the proton gradient of the inner mitochondrial membrane (23, 24). These dyes also induce apoptosis in drug-resistant cancer cells (25, 26) and mediate multimodal tumor therapeutic activities by targeting mitochondria (27, 28). In the present study, we describe the assessment of a mitochondria-targeting small molecule, IR-780, for use as an ICD inducer that can effectively suppress tumor growth and lung metastasis of colorectal cancer (CRC) in a mouse model. IR-780 directly targets mitochondria to destroy cancer cells, including drug-resistant cells, leading to the improved exposure of TAAs and thereby enhancing the effects of ACT, when combined with OT-1 T cell transfer in the B16F10-OVA mouse tumor model. These findings suggest that the mitochondria-targeting small molecule, IR-780, has immense advantages as an ICD inducer, which can mediate tumor-targeted accumulation, full exposure of TAAs, and stimulate strong antitumor immune responses.

## Materials and Methods

### Cell Lines and Animals

Mouse colon cancer cells (CT26) and melanoma cancer cells (B16F10 OVA) were cultured in 1640 medium and DMEM medium with 10% FBS at 37°C with 5% CO_2_. BALB/c and C57BL/6 mice (6 to 8 weeks old) were purchased from the laboratory animal center of the Third Military Medical University. Mice transgenic for a T cell receptor specific for ovalbumine (OT-1) were obtained from the Jackson laboratory and were bred in our SPF laboratory animal room.

### Detection of ICD Markers

CT26 colorectal cancer cells were treated with 10 μM IR-780 for 24 h, then CRT and HSP90 expression levels were determined using an immunofluorescence assay. After washing with PBS, cells were fixed in 4% of paraformaldehyde for 5 min and incubated with anti-calreticulin antibody (Cat# ab2907, Abcam) or anti-Hsp90 antibody (Cat# ab13492, Abcam) at 4°C overnight. After washing with PBS three times, cells were stained with secondary antibody (Cat# ab150077, Abcam) at 37°C for 40 min. Nuclei were stained with DAPI, then observed using fluorescence microscopy. CRT-positive cells were also analyzed by flow cytometry following staining. ATP and HMGB1 released in culture supernatants were detected using an enhanced ATP assay kit (Cat# S0027, Beyotime Biotechnology) and an HMGB1 ELISA kit (Cat# 6010, Chondrex), respectively, according to the manufacturer's instructions.

### Evaluation of DC Maturation *in vitro*

To evaluate DC maturation *in vitro*, bone marrow cells were obtained from BALB/c mice and cultured in complete 1640 medium, supplemented with mouse recombinant macrophage colony stimulating factor (GMCSF, 20 ng/ml, Cat# 576304, Biolegend) and IL-4 (10 ng/ml, Cat# 574304, Biolegend) for 6 days to generate bone marrow-derived DCs. Then, IR-780 treated CT26 cancer cells were co-cultured with the DCs for 24 h. Next, the cells were collected and stained with APC anti-mouse CD11c Antibody (Cat# 117310, Biolegend), Brilliant Violet 421™ FITC anti-mouse CD80 Antibody (Cat# 104706, Biolegend), PE anti-mouse CD86 Antibody (Cat# 105008, Biolegend), or anti-mouse I-A/I-E Antibody (Cat# 107632, Biolegend), and analyzed by flow cytometry.

### Mouse Tumor Models and Treatments

#### Vaccination Assays

The same number of CT26 cells were treated with 10 μM IR-780, 1 μM mitoxantrone (MTX), or 20 μM cisplatin (CIS), respectively for 24 h. After being washed with PBS, 1 × 10^6^ treated cells were injected into the left flanks of each BALB/c mouse (s.c. vaccination site). PBS was injected as the control. One week later, mice were challenged with a subcutaneous injection of 1 × 10^6^ living CT26 cells into the right flanks of mice (re-challenge site). Tumor incidence and growth in each group of mice were routinely monitored for 30 days after the re-challenge. Tumor volumes were calculated as: volume = length × width ^2^ × 0.5.

#### Effects of IR-780 in Tumor Metastasis Inhibition

1 × 10^6^ CT26 cells were treated with 10 μM IR-780 for 24 h and subcutaneously injected into the right flanks of BALB/c mice (IR-780 group). PBS was injected as the control group. After 1 week, each group of mice were injected with 2 × 10^6^ CT26 cells through the tail veins, and then sacrificed at day 20. The tumor sites in the lungs of each group of mice were examined and compared. To investigate immune memory effects, the spleen cells of each group of mice were separated and stained using a Zombie Green™ Fixable Viability Kit (Cat# 423102), CD8a (Cat# 100722), CD44 (Cat# 103040), or CD62L (Cat# 104432) antibodies, and analyzed by flow cytometry. All these antibodies were purchased from Biolegend.

#### Combination Treatment of B16F10-OVA Mouse Tumor Models

B16F10-OVA cells (5 × 10^5^) were suspended in PBS (100 μl) and inoculated subcutaneously into the right flanks of C57BL/6 mice. Four days later, mice were treated with IR-780 (intra-peritoneal, 3 mg/kg) five times, every 2 days. On day 7 after tumor inoculation, mice were injected with OT-1 cells (intra-venous, 2 × 10^6^), which were obtained from OT-1 mice. Cells isolated from tumors and tumor-draining lymph nodes were stained using a Zombie Green™ Fixable Viability Kit and the following fluorochrome-conjugated antibodies: CD45, CD8a, CD69, or KLRG1. For staining of intracellular cytokines (IFN-γ, TNF-α, and Granzyme B), cell suspensions were stimulated with a cell activation cocktail (including Brefeldin A) (Cat# 423303, Biolegend) for 6 h, then fixed and stained with intracellular antigen antibodies (IFN-γ, Granzyme B, and TNF-α), according to the manufacturer's instructions. All antibodies were purchased from Biolegend.

### Statistical Analyses

All the data were presented as mean ± standard deviation. One-way analysis of variance was used to determine significance among groups. Statistical significance was set at ^*^p < 0.05 and ^**^p < 0.01. All the statistical analyses were conducted using the SPSS 13.0 statistical software.

## Results

### Identification of a Tumor-Targeted Small Molecule as a Potential Inducer of ICD

**Figure 1:**
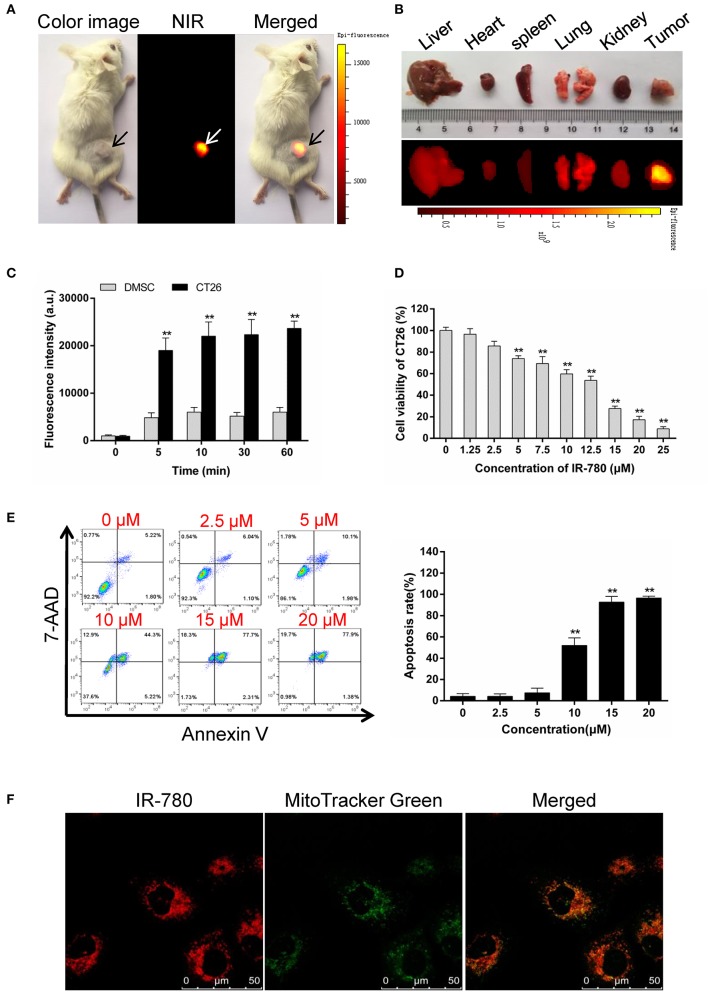
IR-780 selectively accumulates in mitochondria of cancer cells and induces apoptosis. **(A)** Preferential accumulation of IR-780 in the tumor pre-established with CT26 cells. **(B)** The fluorescent imaging of dissected organs. The animal and dissected organs were subjected to imaging with the Kodak *in vivo* FX Pro imaging system. **(C)** NIR fluorescent intensity in mouse dermal mesenchymal stromal cells (DMSCs) and CT26 cells were compared after incubated with 2.0 μM IR-780 for various minutes (*n* = 3). **(D)** CT26 cells viability was tested after treated with different concentration of IR-780 for 24 h (*n* = 5). **(E)** CT26 cells were treated with IR-780 for 24 h and stained with Annexin V/7-AAD to detect cell apoptosis by flow cytometry. **(F)** Co-localization of IR-780 with a mitochondria-specific tracker (Mito Tracker Green) in CT26 cells, imaged using a confocal microscope (scale bars = 50 μm). All the data are presented as mean ± SD. **p < 0.01.

Tumor-targeted ICD inducers stimulate a high level of immune responses in accumulating tumor tissues, which is more necessary and needed for immunotherapy. In our previous study, we found that the near-infrared fluorescent small-molecule, IR-780, could directly target mitochondria in the cancer cells (24), and induce apoptosis in drug-resistant cancer cells (25). Here, we confirmed that IR-780 specifically targeted tumor tissues *in vivo* (Figures 1A,B) and preferentially accumulated in CT26 mouse colorectal cancer cells, relative to normal mouse dermal mesenchymal stromal cells (DMSCs, Figure 1C). IR-780 could efficiently and dose-dependently decrease the viability of CT26 cells (Figure 1D) and induce cell apoptosis (Figure 1E). Moreover, IR-780 exclusively accumulated in the mitochondria of cancer cells and co-localized with the mitochondria-specific fluorescent probe, Mito Tracker Green (Figure 1F), which may help to release mitochondrial antigens and stimulate an efficient antitumor response. All these results indicate that IR-780 may act as a potential inducer of ICD, with tumor-targeting properties and the release of mitochondrial antigens, and could assist in stimulating an antitumor response to kill drug-resistant cancer cells.

### IR-780 Induces ICD and Enhances DC Function *in vitro*

**Figure 2:**
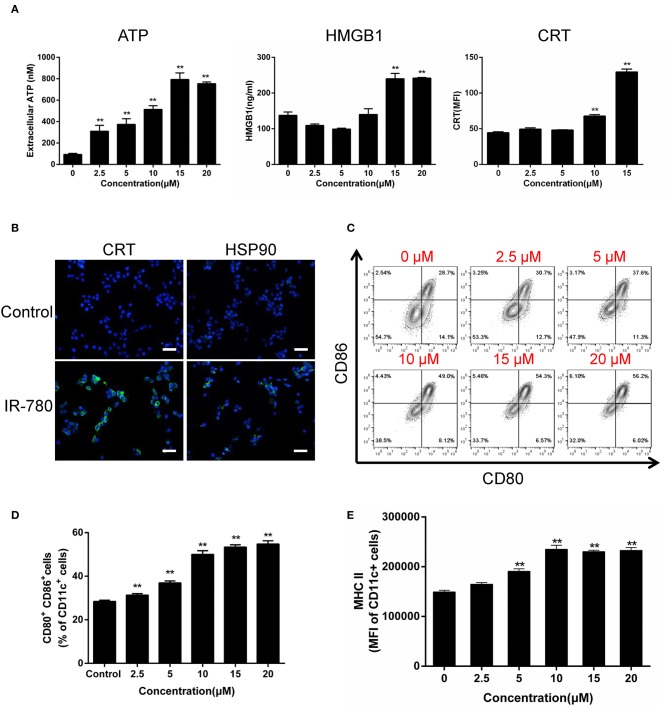
IR-780 induces immunogenic cell death (ICD) *in vitro*. **(A)** Graphical data show the released of ATP, HMGB1, and surface expression of CRT on viable CT26 cells after treated with the indicated concentration of IR-780 (*n* = 3). **(B)** Immunofluorescence detection of CRT and HSP90 expression on the surface of CT26 cells after treated with 10 mM IR-780 for 24 h; scale bars = 20 μm. **(C**, **D)** Flow cytometry analysis of DC maturation by the markers (CD80^+^CD86^+^ of CD11c^+^ cells) after the immature DCs were cultured with IR-780-treated CT26 cells (*n* = 3). **(E)** Flow cytometry analysis the expression of MHCII in the CD11c^+^ cell population after the immature DCs were cultured with IR-780-treated CT26 cells (*n* = 3). All the data are presented as mean ± SD. **p < 0.01.

ICD induces the activation of antitumor immune responses, accompanied by a sequence of changes in the cell surface composition and release of soluble mediators. Here, we tested the hallmarks of ICD in IR-780 treated cancer cells and observed dose-dependent increases in ATP and HMGB1 secretion, as well as the expression of the ER/cytosolic chaperone, CRT, on the surface of cancer cells (Figure 2A). Immunofluorescent assays also indicate cell surface exposure of CRT and the mitochondrial protein, HSP90 (Figure 2B). As DCs have key roles in the recognition of damage associated molecular patterns associated with ICD, and the subsequent uptake and presentation of TAAs, we tested the activation of mouse bone marrow-derived DCs (Supplementary Figure 1) after co-culture with IR-780 treated CT26 cancer cells. IR-780 treatment efficiently increased DC activation and maturation, with up-regulated surface expression of CD80, CD86, and MHCII on DCs (Figures 2C–E). To test whether cancer cell antigen presentation was also enhanced, the peptide antigen transporter gene expression was evaluated, and the results indicated that *Tap1, Tap2, Tapbp, B2m, H2D1*, and *H2K1* mRNA expression levels in cancer cells were increased after IR-780 treatment (Supplementary Figure 2). Altogether, these data clearly demonstrate that IR-780 treatment can induce ICD in cancer cells and increase DC activation and maturation, to enhance the processing and presentation of TAAs.

### IR-780 Induces an ICD Response *in vivo*

**Figure 3:**
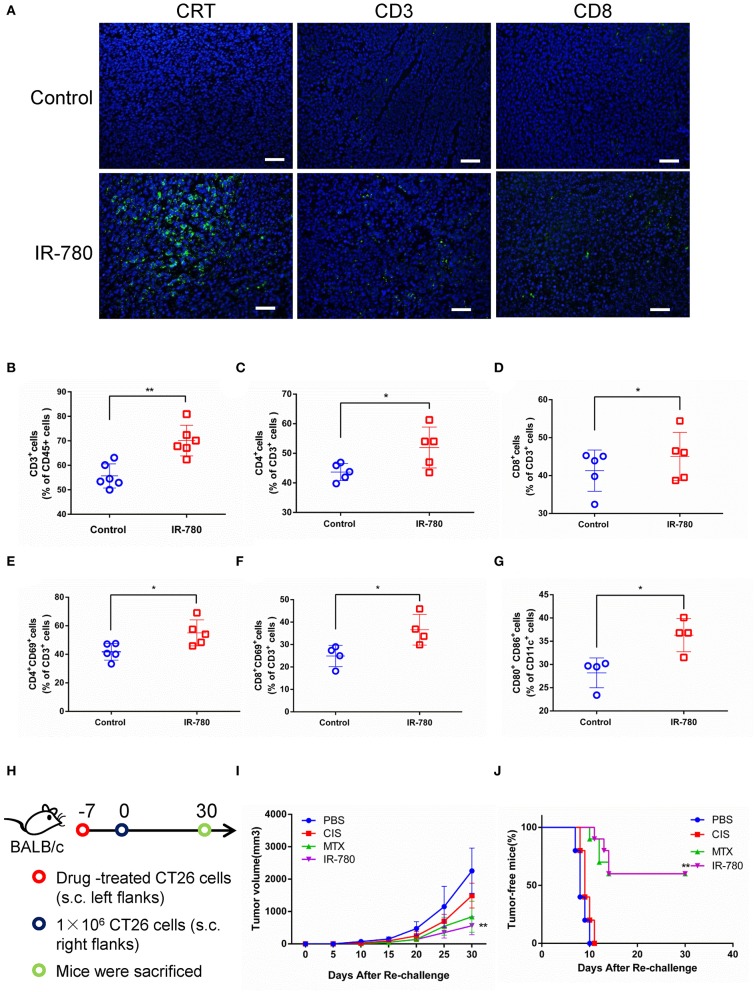
IR-780 induces immunogenic cell death (ICD) *in vivo*. **(A)** Immunofluorescence detection of CRT, CD3, and CD8 expression in tumor sections; scale bar = 20 μm. The percentages of tumor-infiltrating **(B)** CD3^+^, **(C)** CD4^+^, and **(D)** CD8^+^ T cells were analyzed by flow cytometry. And the activation of T cell populations was measured by detecting the percentage of **(E)** CD4^+^CD69^+^ and **(F)** CD8^+^ CD69^+^ T cells populations. **(G)** The activation of CD80^+^CD86^+^ DCs was analyzed by flow cytometry. **(H)** Schedule representation of different treatment-mediated antitumor vaccination effects in CT26 tumor models. **(I)** After subcutaneous injection of different treated CT26 cells (10 μM IR-780, 1 μM MTX, and 20 μM CIS, respectively) in the left flank of mice, the tumor volumes in the right flank of each group mice were detected and compared (tumor volume = length × width ^2^ × 0.5). **(J)** The frequency of tumor-free mice in each group after vaccinated with different pretreated CT26 cells (*n* = 10). All the data are presented as mean ± SD. *p < 0.05; **p < 0.01 as comparing with control group.

To discriminate between ICD and non-ICD inducers, a systematic analysis of the results of vaccination of immunocompetent and antitumor adaptive immune responses in syngeneic mice treated with dying cancer cells is the gold standard for determining ICD induction (29). In this study, IR-780 was intraperitoneally injected into BALB/c mice bearing CT26 tumors, to evaluate the adaptive immune response *in vivo*. IR-780 effectively inhibited tumor growth, increased the expression of CRT on cancer cells, and increased T cell infiltration into tumor tissue (Figure 3A). On day 10, after treatment with IR-780, tumors were harvested and analyzed by flow cytometry. Compared to the control group, we found that IR-780 treatment clearly increased the infiltration of both CD4^+^ and CD8^+^ T cells into tumor tissues (Figures 3B–D). Additionally, a high proportion of tumor-infiltrating T cells in the treatment groups expressed CD69, a marker of T cell activation (Figures 3E,F). Moreover, IR-780 treatment also increased the number of activated CD11c^+^ DCs infiltrating tumor tissues, which are important for the stimulation of tumor cell-specific immune responses (Figure 3G). Further, we studied the immunogenic potential of IR-780 treatment in the context of a mouse vaccination model. We treated CT26 cancer cells with PBS, CIS (proven not to induce an ICD response), MTX (proven to induce ICD) (30), and IR-780 *in vitro* and then injected them into the left flank of immunocompetent BALB/c mice. Mice were re-challenged with live CT26 cancer cells by subcutaneous (s.c.) injection into the right flank at day 7 (Figure 3H). Tumor growth and tumor-free survival were measured and compared among mice (Figures 3I,J). All these results clearly establish that IR-780 acts as an ICD inducer *in vivo*.

### IR-780 Effectively Suppresses Tumor Metastasis in a CRC Mouse Model

**Figure 4:**
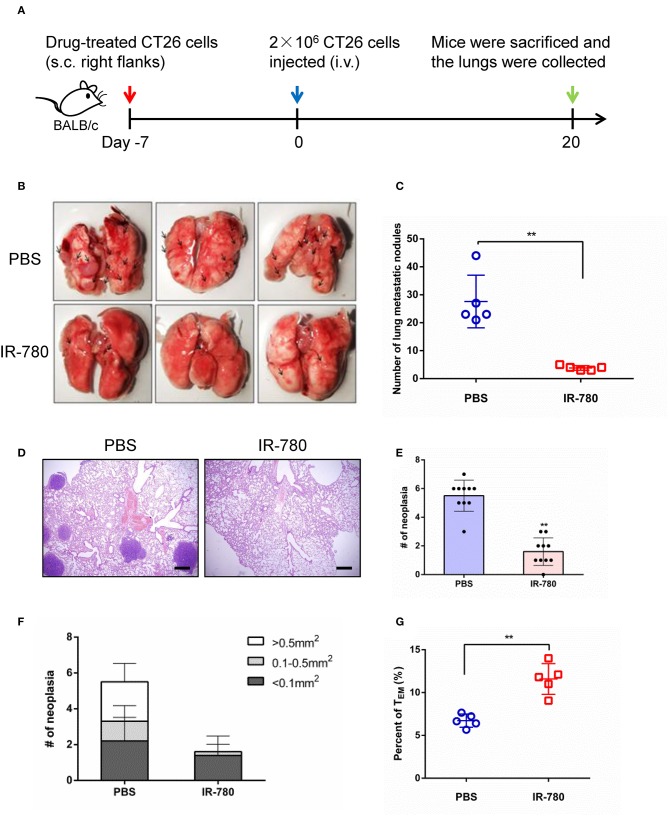
Tumor metastasis prevention effects via IR-780 induced long-term immune memory. **(A)** Therapeutic schedule for IR-780 mediated inhibition of tumor metastasis in mouse model. **(B)** Representative photographs of lung tissue in different group mice. **(C)** Quantification of pulmonary metastasis nodules on control mice or mice pre-vaccinated with IR-780 treated CT26 cancer cells. **(D)** H&E staining of the lung tissue collected at day 20; Scale bars = 200 μm. **(E**, **F)** Quantification of pulmonary metastasis neoplasia and tumor sizes on pulmonary sections. **(G)** The proportions of effector memory T cells (CD8^+^CD44^+^CD62L^−^) in the spleen of different group mice detected by flow cytometry. All the data are presented as mean ± SD. **p < 0.01.

We next assessed the immunotherapeutic effects of IR-780 on tumor metastasis in a mouse model. CT26 cancer cells were treated with IR-780 *in vitro* and then injected (s.c.) into the right flank of immunocompetent BALB/c mice. Then, 2 × 10^6^ live CT26 cancer cells were injected into the mouse tail veins at day 7. Twenty days after injection, mice were sacrificed, and their lungs collected, and metastatic foci analyzed (Figure 4A). Mice vaccinated with IR-780 treated CT26 cells had significantly less tumor metastases in the lungs, relative to the control group (Figures 4B,C). Hematoxylin and eosin staining of lung tissues also indicated that vaccination with IR-780 treated CT26 cells led to decreased tumor metastasis, with fewer and smaller metastatic lesions in the lungs, relative to the control group (Figures 4D–F). To verify that IR-780 treatment can induce a systemic immune response to inhibit distant tumor metastasis and generate immune-memory effects, spleen effector memory T cells (CD8^+^ CD44^+^ CD62L^−^) were analyzed by flow cytometry. Vaccination with IR-780 treated CT26 cells led to a significantly increased frequency of effector memory T cells (Figure 4G), suggesting that a memory immune response was established in mice treated with IR-780.

### IR-780 Enhances the Effects of ACT in the B16F10-OVA Mouse Tumor Model

**Figure 5:**
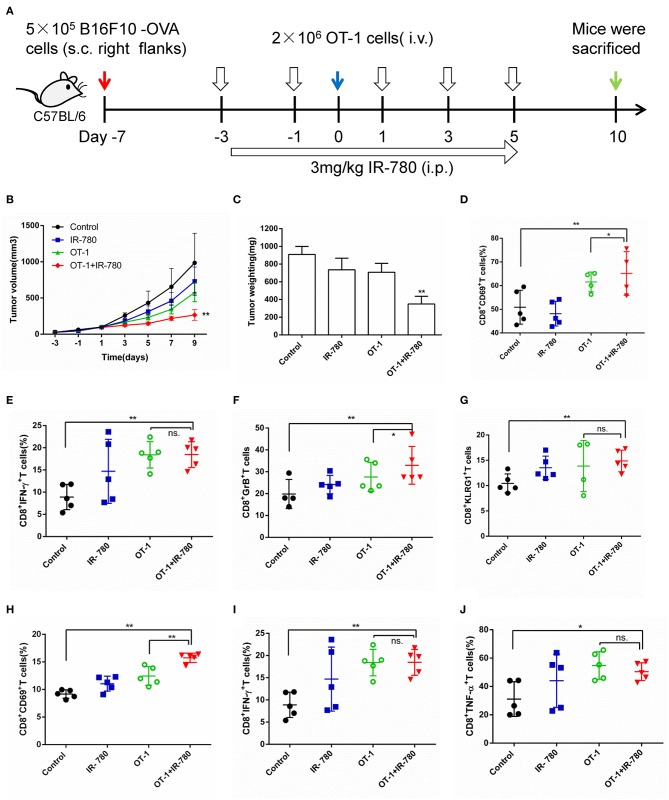
Combinatorial treatment of IR-780 with OT-1 cells therapy in B16F10-OVA tumor mouse models. **(A)** Therapeutic schedule for combination therapy in mouse models. **(B)** The tumor volumes were detected every 2 days in different groups (volume = length × width^2^ × 0.5; *n* = 8). **(C)** The tumor weight in different groups at day 10. The percentage of activation of CD8^+^ T cells in tumors **(D)** and tumor-draining lymph nodes **(H)**. Flow cytometry analysis of the functional CD8^+^ T cells **(E)** IFN-γ ^+^, **(F)** GzB^+^, and **(G)** KLRG1^+^ cells in tumor tissue, and **(I)** IFN-γ^+^, **(J)** TNF-α^+^ cells in tumor-draining lymph nodes. All the data are presented as mean ±SD. *p < 0.05; **p < 0.01.

Given the great advantages of IR-780 in tumor targeting and eliciting antitumor immune responses, it was combined with ACT to evaluate its effects in this context. First, CT26 tumor mouse models were treated using a combination of IR-780 and T cells [mouse spleen T cells stimulated using plate-bound anti-CD3 and IL-2, and soluble anti-CD28, for 4 days (31)]. The results indicated that IR-780 significantly enhanced the antitumor effects of T cells in the CT26 tumor model (Supplementary Figure 3). Next, a B16F10-OVA mouse tumor model was established by s.c. injection of the B16F10-OVA melanoma cell line (5 × 10^5^ cells), which stably expresses chicken ovalbumin, in the right flanks of mice. Cognate peptide-activated OT-1 T cells, designed to recognize B16F10-OVA cells, were used alone, or combined with IR-780, to treat B16F10-OVA tumor model mice (Figure 5A). OT-1 T cell treatment (2 × 10^6^ CD8^+^ Vα2^+^ cells, Supplementary Figure 4) exhibited a minor therapeutic impact in this model, indicating the substantial negative effect of immunosuppression in the TME. In contrast, combination treatment with 3.0 mg/kg IR-780 and OT-1 T cells significantly reduced tumor growth (Figures 5B,C) without induced obvious toxicity in mice (Supplementary Figure 5). Further, on day 10 of combination treatment, the number of activated (CD8^+^ CD69^+^) T cells in the tumor were increased (Figure 5D) and T-cell functions were enhanced, with an up-regulated expression of the effector T-cell markers, IFN-γ, Granzyme B, and KLRG1 (Figures 5E–G). We also stained for intracellular expression of CD69, IFN-γ, and TNF-α in inguinal lymph node CD8^+^ T cells. Combined treatment induced higher levels of activated CD8^+^ T cells in the lymph nodes, leading to an improved therapeutic outcome (Figures 5H–J). Further evidence that IR-780 induced a more pronounced effector phenotype was attained by the assessment of multiple CTL effector markers through real-time qPCR of the whole tumor mRNA, demonstrating increased expression of *IFN-*γ, *TNF-*α, *Granzyme B, EOMES, Perforin, BLIMP-1, FOXO3*, and *CXCL10* (Supplementary Figure 6). All these data indicate that a combined treatment strategy, including the tumor-targeted ICD inducer, IR-780, increased tumor infiltration of T cells and their function, and significantly enhanced the effects of ACT in solid tumors.

## Discussion

ACT involves the infusion of autologous or non-autologous lymphocytes into patients after expansion *ex vivo*. Given its remarkable early results, the Food and Drug Administration has approved ACT for certain types of tumors. ACT has recently been adapted to use gene-modified T cells, with both TCR- and CAR-based therapy achieving tremendous success against B cell malignancies (32); however, this approach has been less successful against solid cancer, partly due to the immunosuppressive TME (4, 33). The TME comprises a stromal compartment, which helps to establish the appropriate network required to support tumor development, including immunosuppressive cells, such as regulatory T cells, myeloid-derived suppressor cells, tumor-associated macrophages, neutrophils, and stromal fibroblasts, which secrete immunosuppressive factors and up-regulate inhibitory ligands, including PD-L1, which can suppress the effectiveness of ACT (34–36). TMEs are also characterized by T cell exhaustion, resulting from chronic, but suboptimal, exposure to TAAs, without sufficient co-stimulation (37). Excitingly, with the growth of knowledge of immune cells and their interaction with the TME, newly developed combinatorial strategies have allowed us to identify key requirements for overcoming the challenges imposed by the immunosuppressive TME. Combination strategies that promote ACT antitumor responses, persistence, and trafficking, will greatly improve therapeutic effects in solid tumors. The main combinatorial strategies used in ACT include targeting immune checkpoints, using immune agonists to boost immune responses in ACT, regulating the cytokine and/or chemokine milieu to enhance ACT, and enhancing T cell activity against solid tumors by vaccine boosting. These approaches have demonstrated tremendous potential in various preclinical models and are in the process of being translated to the clinic (38).

ICD can convert dying cancer cells into a therapeutic vaccination and induce the release of key immunostimulatory factors or signals to drive efficient antitumor immune responses (39, 40). Therefore, ICD inducers are widely-studied in clinical practice and are often used in combinatorial approaches aimed at subverting the highly immunosuppressive TME of solid tumors, since the induction of ICD can facilitate an initial T cell-driven anticancer immunity by resetting the TME to favor T cell infiltration. Combination approaches, using an ICD inducer alongside checkpoint blockade immunotherapies, are widely demonstrated in preclinical models as having superior benefits against various malignant tumors. In addition, ICD inducers can be used as neoadjuvants to increase tumor-infiltrating lymphocytes in tumor tissues, which can serve as a source of T cells for subsequent ACT (41, 42). Moreover, ICD inducers can be administered following ACT, to re-stimulate the effective antitumor activity of ACT cells, by fully exposing TAAs. While applications of ICD inducers combined with immunotherapy are widely reported to exhibit superior performance, how optimal therapeutic outcomes can be achieved using ICD inducers remains to be determined, particularly given the limited intra-tumoral accumulation and inadequate circulation half-life time of conventional ICD inducers, as well as their induction of off-tumor inflammatory responses (14, 43). To address these challenges, strategies to develop tumor-targeted ICD inducers have the potential to provide safe and effective approaches for use in combination immunotherapy (44). Given the important roles of mitochondria in tumorigenesis and tumor progression, and the recognition of mitochondrial damage as a hallmark of cancer, in this study we assessed the ICD inducer, IR-780, which targets cancer cell mitochondria. IR-780 specifically accumulates in tumor cells to elicit ICD *in vitro* and *in vivo* and effectively suppresses the growth of tumors and lung metastases in a CRC mouse model. Specifically, IR-780 directly targets mitochondria to destroy cancer cells, including drug-resistant cancer cells, which can lead to complete exposure of TAAs, including tumor-specific mitochondrial antigens. IR-780 significantly enhanced the effects ACT in the B16F10-OVA mouse tumor model, when combined with OT-1 T cell transfer.

In conclusion, we present a new strategy for the development of tumor-targeted ICD inducers, by targeting tumor mitochondria and demonstrate its application in combination with ACT for the treatment of solid tumors. Our combination immunotherapy approach achieved potent antitumor efficacy, leading to the significant inhibition of solid tumors in animal models, representing a valuable reference in the field of tumor-targeted immunotherapy.

## Data Availability Statement

The datasets generated for this study are available on request to the corresponding author.

## Ethics Statement

The animal study was reviewed and approved by Animal Care and Use Committee Guidelines of the Third Military Medical University.

## Author Contributions

QJ, CZ, and HW performed the experiments. QJ and CZ interpreted data and drafted the manuscript. TP and LZ performed the data analysis. YW, WH, and CS conceived and designed the study and wrote the manuscript.

### Conflict of Interest

The authors declare that the research was conducted in the absence of any commercial or financial relationships that could be construed as a potential conflict of interest.
