# Supplementary material for: Mitochondria-Targeting Immunogenic Cell Death Inducer Improves the Adoptive T-Cell Therapy Against Solid Tumor
Source: Front Oncol. 2019 Nov 12;9:1196. doi: 10.3389/fonc.2019.01196 (PMC6861368; doi:10.3389/fonc.2019.01196)
Supplement: Supplementary file 1 [file Table_1.DOC]

Supplementary Material

# Supplementary Figures

**
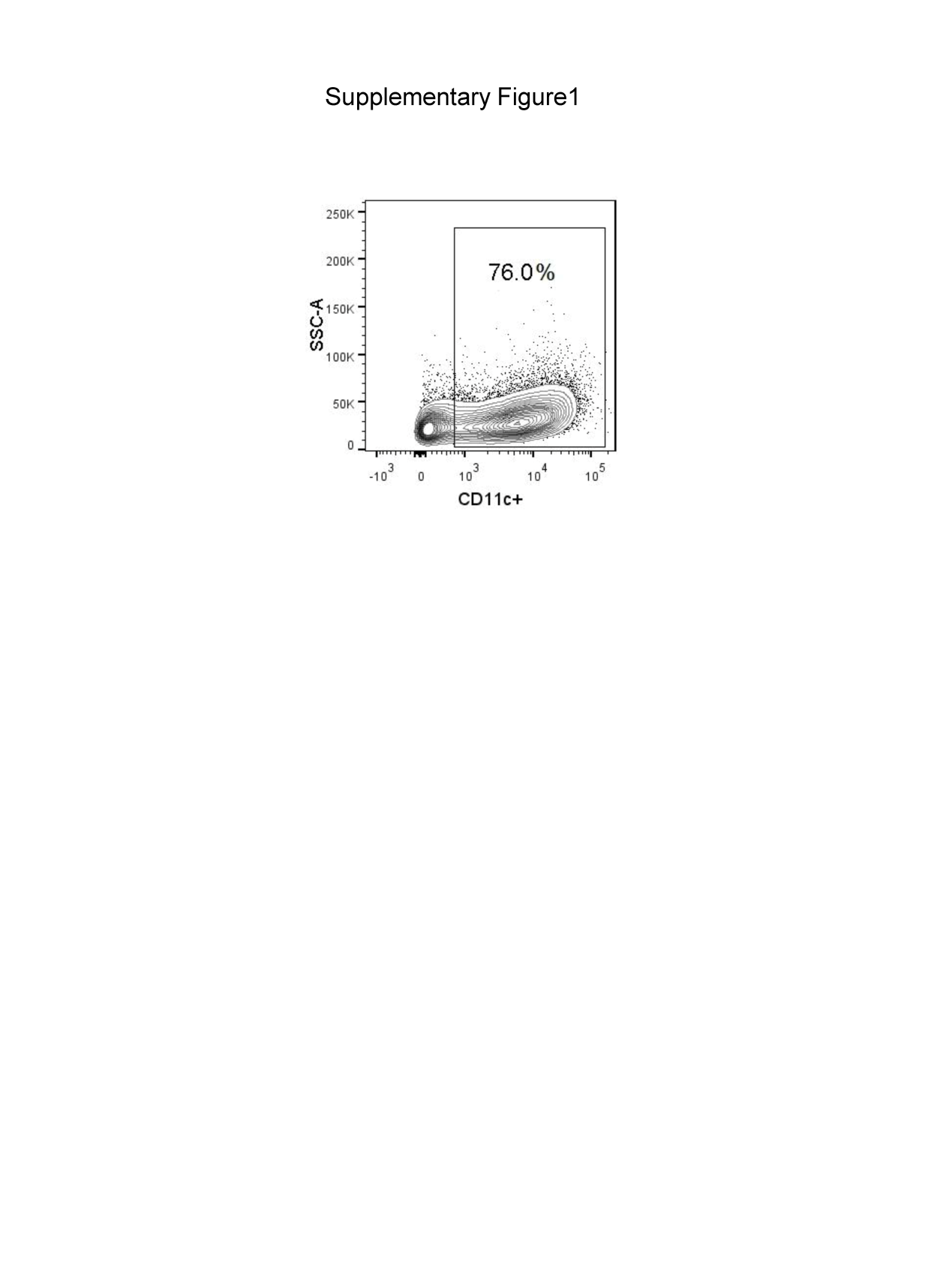
**

**Supplementary Figure 1 |** The percentage of CD11c^+^ cells in mouse bone marrow-derived DCs after 6 days induction.


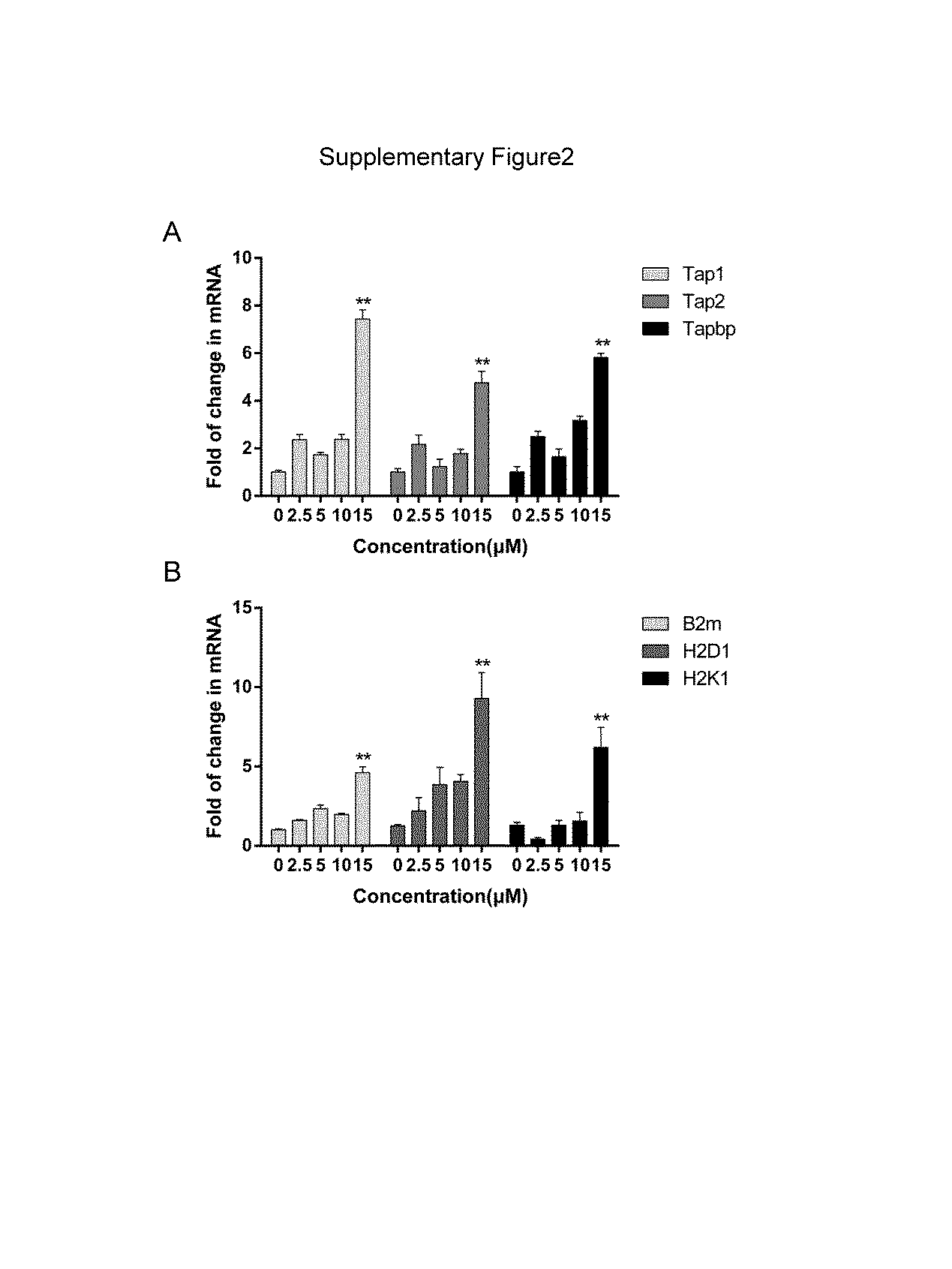


**Supplementary Figure 2 |** Real-time qPCR detection of indicated antigen presentation genes expression in CT26 cells after treated with different concentration of IR-780 for 24h (n=3; **p<0.01).


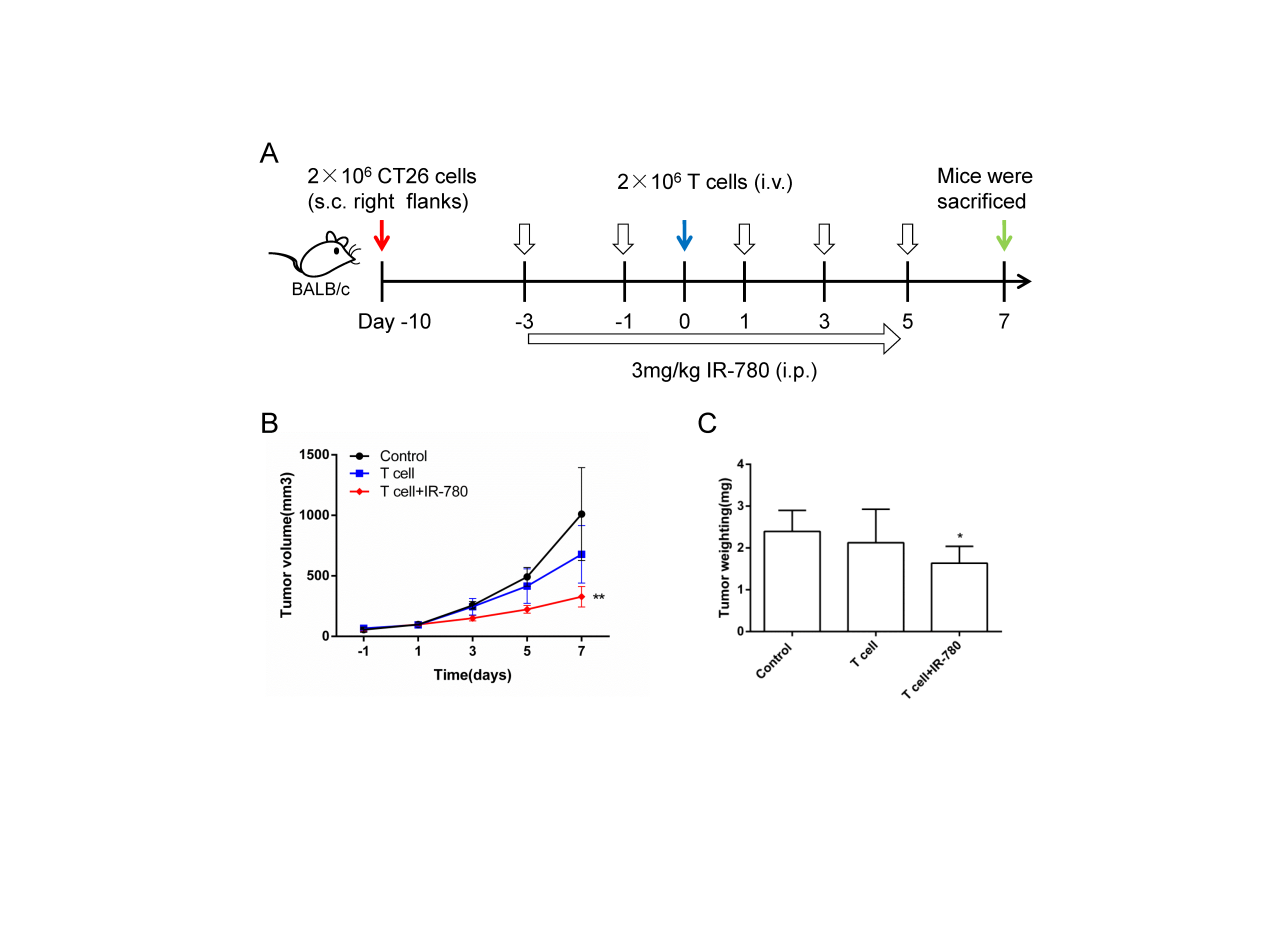


**Supplementary Figure 3 |** Combinatorial treatment of IR-780 and adoptive T cells therapy inhibits tumor growth in CT26 tumor models. **(A)** Therapeutic schedule of IR-780 and adoptive T cells combination therapy. **(B)** The tumor volumes were detected every two days in different groups (volume = length × width ^2^ × 0.5). **(C)** The tumor weight in different groups at day 7 (n = 8). All the data are presented as mean ± SD. *p < 0.05.


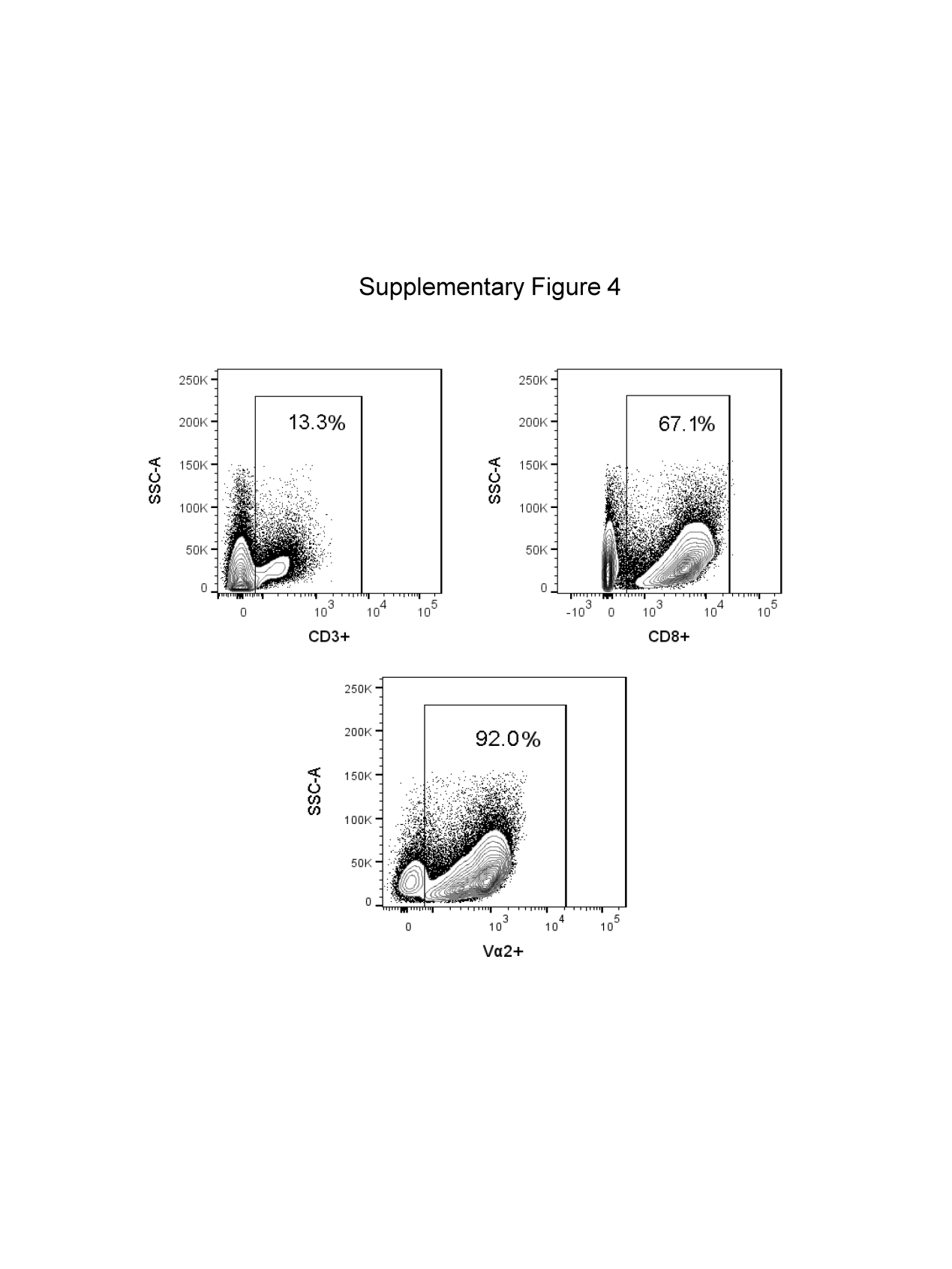


**Supplementary Figure 4 |** The percentage of CD8^+^ OT-1^+^ T cells which was isolated from the spleen of OT-1 transgenic mice.


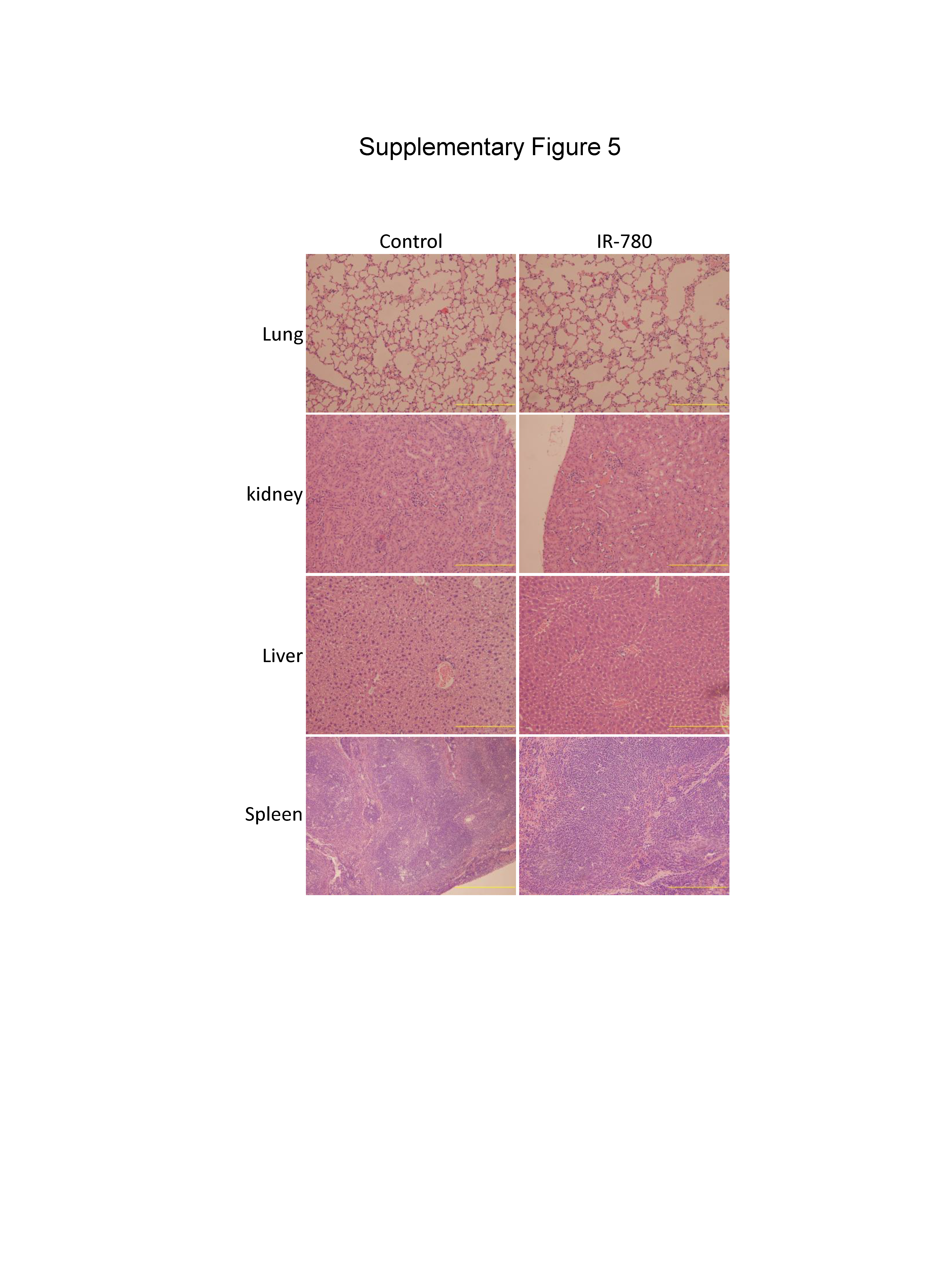


**Supplementary Figure 5 |** Histopathologic analysis of vital organs in mice after treated with IR-780, the figure indicated no obvious changes in control and treatment mice.


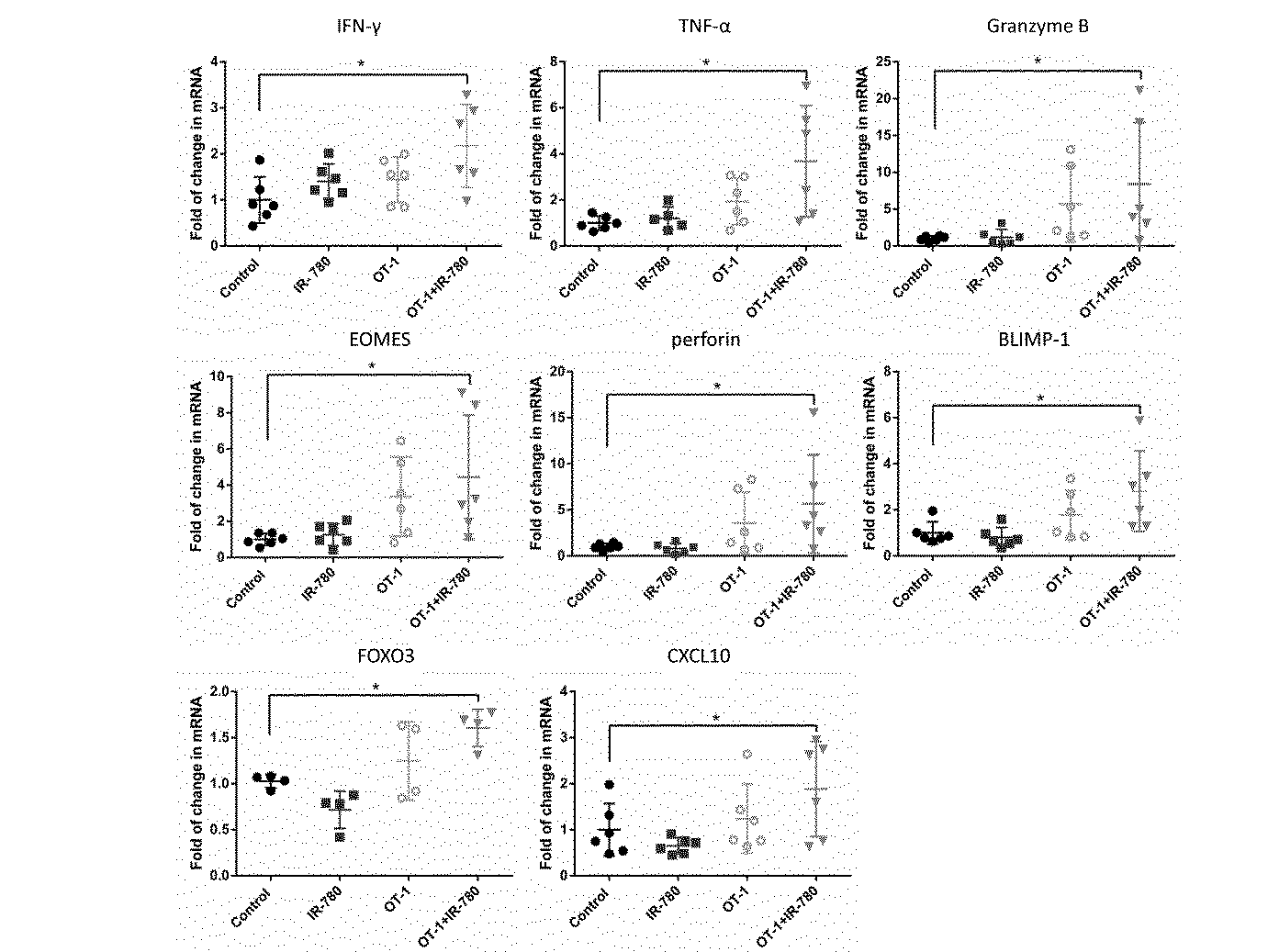


**Supplementary Figure 6 |** Real-time qPCR detection of indicated gene expression on the whole tumors from different groups of mice. All the data are presented as mean ± SD. *p < 0.05.
